# Considerations on the Taxonomy of the Genus *Arhuaco* Adams and Bernard 1977, and its Relationships with the Genus *Pronophila* Doubleday [1849] (Nymphalidae, Satyrinae)

**DOI:** 10.1007/s13744-018-0641-4

**Published:** 2018-11-10

**Authors:** T W Pyrcz, J Lorenc-Brudecka, A Zubek, C Prieto, P Boyer, K Florczyk, B Wacławik, D Lachowska-Cierlik

**Affiliations:** 1 Nature Education Centre, Jagiellonian Univ, Gronostajowa 5, 30-387 Kraków, Poland; 2 Entomology Dept, Institute of Zoology and Biomedical Research, Jagiellonian Univ, Kraków, Poland; 3 Depto de Biología, Universidad del Atlántico, Barranquilla, Colombia; 4 SNSB-Bavarian State Collection of Zoology, Münchhausenstrasse 21, 81247 Munich, Germany; 5 7 Lotissement l’Horizon, Le Puy Sainte Réparade, France

**Keywords:** *Arhuaco*, cloud forests, Costa Rica, Sierra Nevada de Santa Marta, *Pronophila*, Pronophilina, COI, RpS5

## Abstract

*Arhuaco* Adams & Bernard (1977) is one of the least known genera of Neotropical Satyrinae. It comprises two species and presents an unusual disjunct distribution, with *A. ica* Adams & Bernard (1977), endemic to the isolated Colombian Sierra Nevada de Santa Marta, and *A. dryadina* (Schaus 1913) found in the mountains of Costa Rica and Panama. Here, the female of *A. dryadina* is described, and a new generic diagnosis is presented. Affinities with other genera of the subtribe Pronophilina, in particular the potential closest relatives, such as *Pronophila* Doubleday (1849), are investigated based on morphological, molecular, ecological, and behavioral data. Results from molecular and morphological sources are incongruent. Molecular data indicate that *Arhuaco* is paraphyletic, with *A. dryadina* segregating within the *Pronophila* clade. Morphological data, by contrast, indicate a closer affinity between the two species currently placed in *Arhuaco*, favoring the monophyly of the genus, and show no consistent synapomorphies for *Arhuaco* + *Pronophila*. A vicariance biogeographical scenario is evaluated.

## Introduction

Research on Neotropical satyrines (Nymphalidae, Satyrinae) has intensified during the last decade, and a number of valuable contributions have been published on their relationships and taxonomy, using molecular, morphological, early-stage, and ecological data, vastly expanding our knowledge on the evolution of this group of butterflies, and in particular of the montane genera (e.g., Peña *et al*^2011^, Pyrcz *et al*^2009^, Casner & Pyrcz 2010, Marín *et al*^2017^, Pyrcz *et al*^2017^). However, the two subtribes Pronophilina and Euptychiina, which constitute the bulk of Neotropical Satyrinae, are extremely diverse with well over 1000 species and at least 100 genera (Lamas 2004, Pyrcz 2010), and several genera have still not been thoroughly investigated.

One of these is *Arhuaco* Adams & Bernard 1977, arguably one of the least studied and most intriguing genera within the subtribe Pronophilina. It contains, as currently recognized, two species: *A. ica* Adams & Bernard 1977, endemic to the isolated northern Colombian Sierra Nevada de Santa Marta, and *A. dryadina* (Schaus 1913) confined to the Mesoamerican ranges of Talamanca and Meseta Central in Costa Rica and western Panama. The latter species was described originally in the catch-all genus *Catargynnis* Röber, 1892, later synonymized as a junior synonym of *Pseudomaniola* Röber, [1889] (Adams 1986).

The available information on *Arhuaco* is scanty. Adams and Bernard (1977) described the genus for the new species *Arhuaco ica* Adams & Bernard, based on male adult morphology, with reference to head parts, wing shape, androconia, color pattern, and male genitalia. Pyrcz (1999) examined the collection of Edwin Krüger and noticed that this German collector found both sexes of *A. ica* some 50 years before Adams and Bernard, but for some reason did not mention it in any of his papers dedicated to Colombian Satyrinae (Krüger 1924, 1925). Pyrcz (op. cit.) also described the female of *A. ica*. *Catargynnis dryadina* was described by Schaus (1913), and was later listed by Gaede (1931) and illustrated by D’Abrera (1988). The only comment on the behavior and ecology of this species was by De Vries (1987). Pyrcz (2004) transferred *C. dryadina* to the genus *Arhuaco* based on similarities in the male adult morphology in comparison with *A. ica*.

Both *A. ica* and *A. dryadina* are considered very rare in the field (Adams & Bernard 1977, De Vries 1987), which is reflected in the scarcity of available material in scientific collections. *Arhuaco ica* is known only from a couple of specimens collected by Edwin Krüger in 1919 and 1925, by Michael Adams and George Bernard in 1972 and 1975, and by C. Gibson in 1974 (Adams & Bernard 1977), and recently from two males collected by Carlos Prieto in 2011 and 2013. The only known female is that collected by Krüger. The meagerness of data on *A. ica* is perhaps not surprising since the Sierra Nevada de Santa Marta, although easily reachable from the coast, has been for many years nearly inaccessible due to political unrest. The scarcity of *A. dryadina* in scientific collections is, however, less understandable. This species occurs in central Costa Rica in the vicinity of large towns, and in areas reachable by asphalt roads. Moreover, the butterfly fauna of Costa Rica is considered as arguably the best investigated among all Central and South American countries (De Vries 1987, 1997, Chacón & Montero 2007).

To date, only the external morphology of the male of *A. dryadina*, male and female *A. ica*, and male genitalia of the latter species has been examined. The female of *A. dryadina* was unknown prior to this study. No molecular data have been published for either species. Thus, opportunities for more rigorous systematic studies have been extremely limited, and the validity of *Arhuaco* is based exclusively on a comparison of wing shape and color pattern, in particular the configuration of ventral groundplan elements sensu Nijhout (1991). In order to more reliably evaluate the monophyly of *Arhuaco*, therefore, both male and female genitalia were examined and compared with other genera of Pronophilina, and genetic distances were estimated in order to better establish the systematic position of the two species within the subtribe.

*Arhuaco* is also particularly intriguing because of its disjunct distribution, which is unique among the cloud forest members of the Neotropical subtribes Pronophilina and Euptychiina. There is no other genus which shows such an atypical pattern, with species found in vastly separated geographical areas. Other satyrine genera occurring in the cloud forests of Central America are either endemic to that region (*Drucina* Butler 1872, *Cyllopsis* R. Felder 1869) or widespread throughout the Andes (*Oxeoschistus* Butler 1867, *Pedaliodes* Butler 1867, *Eretris* Thieme 1905, and *Lymanopoda* Westwood 1851). Conversely, the genera found in the Sierra Nevada de Santa Marta are either endemic (*Paramo* Adams & Bernard 1977, *Sierrasteroma* Adams & Bernard 1977, the latter, however, synonymized with *Steroma* Westwood [1850] (Pyrcz 2010)) or widespread (*Eretris* Thieme 1905, *Pedaliodes* Butler 1867, *Lymanopoda* Westwood 1851, and *Corades* Hewitson 1849). Comparative studies carried out here are therefore expected to shed some light on the possible origins of *Arhuaco*.

## Materials and Methods

### Morphology

Male and female abdomens of *A. ica*, *A. dryadina*, and several other species of Pronophilina, as listed in the “Material examined” section, were removed and soaked in 10% KOH solution for 5–10 min. Subsequently, the abdomens were preliminarily cleaned of soft tissue in water in order to expose genital parts. Female abdomens were stained in chlorazole black to better visualize weakly sclerotized structures. Dissected genitalia were subsequently cleaned in ethanol 90% and 95% solutions. A Nikon digital camera DS-Fi1 and an Olympus SZX9 stereomicroscope were used to take pictures of dissections, and images were then processed in Combine ZP and Corel PHOTO-PAINT X3 programs to enhance focus and improve quality. Genital dissections were kept in glycerol vials pinned under corresponding specimens. Genital terminology follows largely Klots (1956). Wing venation pattern and other external macrostructures were examined under a stereomicroscope Delta Optical SZ-630. The following collection acronyms are used: CEP-MZUJ: Nature Education Centre (formerly Zoological Museum) of the Jagiellonian University, Kraków, Poland; MIZPAN: Museum and Institute of Zoology, Polish Academy of Sciences, Warsaw, Poland; NHML: Natural History Museum, London, UK; RCCP: Research Collection of Carlos Prieto, Cali, Colombia; NMNH: National Museum of Natural History, Smithsonian Institution, Washington DC, USA.

### Molecular analysis

For the DNA analysis, single legs of *A. ica*; *A. dryadina*; *Lasiophila regia* Staudinger, 1897; *Pronophila thelebe* Doubleday [1849]; *Pronophila unifasciata* Lathy, 1906; *Pronophila timanthes* Salvin, 1871; and *Pseudomaniola gerlinda* (Thieme, 1907) were removed. Additionally, sequences for six species were imported from GenBank: *Lasiophila cirta* C. Felder & R. Felder, 1859; *Oxeoschistus leucospilos* Studinger, 1876; *Oxeoschistus pronax* (Hewitson, 1850); *Mygona irmina* (Doubleday 1849); *Junea dorinda* (C. Felder & R. Felder, 1862); and *Lymanopoda obsoleta* (Westwood 1851) as an outgroup. DNA was isolated using a Sherlock AX (A&A Biotechnology) extraction kit. Amplification of part of the mitochondrial gene *COI* and the nuclear gene *RpS5* was done using the following pairs of primers, respectively: LCO1490 and HCO2198 (Folmer *et al*^1994^), HybrpS5degF and HybrpS5degR (Wahlberg and Wheat 2008), with standard PCR protocol. The PCR fragments of 569 bp (*COI*) and 526 bp (RpS5) were sequenced using a BigDye Terminator v.3.1. Cycle Sequencing Kit (Applied Biosystems, Foster City, CA, USA) and ran on an ABI 3100 Automated Capillary DNA Sequencer. All newly obtained sequences were deposited in GenBank (accession numbers are provided in Table 1). Sequences for each marker were aligned in MAFFT version 7 (Katoh & Toh 2008) using the default settings, and manually checked against non-conservative alignments in BioEdit 5.0.0. (Hall 1999). Uncorrected pairwise distances were calculated in MEGA version 7 (Kumar *et al*^2016^). A maximum likelihood (ML) topology for *COI*, *RpS5*, and concatenated sequences was constructed using RAxML v8.0.19 (Stamatakis 2014). The strength of support for internal nodes of the ML tree was measured using 1000 rapid bootstrap replicates. All trees were visualized in FigTree v. 1.4.3 (http://tree.bio.ed.ac.uk/software/figtree).

**Table 1:** Genetic pairwise distances between analyzed *COI* sequences.

*Mygona irmina_*KU359854												
*Junea dorinda*_KU359876	0.111											
*Lasiophila cirta*_DQ338851	0.097	0.104										
*Oxeoschistus leucospilos*_DQ338854	0.104	0.114	0.091									
*Oxeoschistus pronax*_GQ357235	0.121	0.125	0.109	0.074								
*Lymanopoda obsoleta*_KU359892	0.135	0.114	0.114	0.130	0.141							
*Pronophila thelebe*	0.086	0.093	0.088	0.112	0.123	0.105						
*Pronophila unifasciata*	0.091	0.093	0.091	0.112	0.120	0.104	0.053					
*Pronophila timanthes*	0.109	0.097	0.098	0.121	0.134	0.105	0.067	0.039				
*Arhuaco dryadina*	0.112	0.104	0.111	0.132	0.132	0.118	0.065	0.067	0.076			
*Pseudomaniola gerlinda*	0.120	0.139	0.100	0.120	0.123	0.125	0.116	0.102	0.112	0.114		
*Lasiophila regia*	0.091	0.100	0.056	0.091	0.105	0.102	0.086	0.083	0.093	0.107	0.095	
*Arhuaco ica*	0.086	0.084	0.077	0.105	0.120	0.105	0.065	0.033	0.054	0.076	0.107	0.069

### Material examined

***Arhuaco dryadina***: Holotype (♂): Turrialba, 8200 ft., Aug., Type no. 16781, U.S.N.M, NMNH; 6 ♂ and 4 ♀: Costa Rica, Cordillera de Talamanca, Cerro de la Muerte, 2950–3000 m, 01-03.VII.2015, 9°32′30″N, 83°43′14″W, T. Pyrcz *leg.* (DNA extraction numbers AZ–105, AZ–106; 1 ♂: prep. genit. 149/30.09.2015 J. Lorenc-Brudecka, CEP-MZUJ; 1 ♀: prep. genit. 41/15.07.2015 J. Lorenc-Brudecka); ***Arhuaco ica***: Holotype (♂): Colombia, Sierra Nevada de Santa Marta, East of San Pedro, 2700 m, 06.VIII.1972, M. Adams *leg.*, NHML; 1 ♂: Sierra Nevada de Santa Marta, San Pedro, 2400 m, 14.I.2013, 10°53′N, 73°58′W, i813, C. Prieto *leg.*, RCCP, DNA extract number AZ–212; 1 ♂: Sierra Nevada de Santa Marta, San Pedro, 2400 m, 11.I.2011, 10°53′N, 73°58′W, i748, C. Prieto *leg.*, RCCP; 1 ♂: Sierra Nevada de Santa Marta (no exact locality), 2400 m, 27.IX.1919, E. Krüger *leg.*, prep. genit. 150/30.09.2015 J. Lorenc-Brudecka, MIZPAN; 1 ♀: Sierra Nevada de Santa Marta (no exact locality), 2400 m, 24.VII.1925, E. Krüger *leg.*, prep. genit. 148/29.09.2015 J. Lorenc-Brudecka), MIZPAN; ***Pronophila timanthes***: 1 ♀: Costa Rica, Cerro de la Muerte, División – Santa Eduviges, 1900–2050 m, 19.III.2016, 9°29′41″N, 83°44′04″W, T. Pyrcz *leg.*, prep. genit. 489/22.11.2016 J. Lorenc-Brudecka, CEP-MZUJ; 1 ♂: Costa Rica, Cerro de la Muerte, División – Santa Eduviges, 1700–1850 m, 04.III.2016, T. Pyrcz *leg.*, MZUJ, prep. genit. 518/15.03.2017 J. Lorenc-Brudecka; 1 ♂: Costa Rica, Cerro de la Muerte, División – Santa Eduviges, 1700–1850 m, 09.III.2016, T. Pyrcz *leg.*, DNA extraction number AZ–249, CEP-MZUJ; 1 ♂: Costa Rica, Cerro de la Muerte, División – Santa Eduviges, 1900–2050 m, 14.III.2016, T. Pyrcz *leg.*, CEP-MZUJ, DNA extraction number AZ–248; ***Pronophila rosenbergi puyango*** Pyrcz, 2000: 1 ♀: Peru, Amazonas, Cocabamba, 2000 m, II.2002, B. Calderón *leg.*, prep. genit. 03/14.04.2016 K. Florczyk, CEP-MZUJ; ***Pronophila obscura*** Butler, 1868: 1 ♀: Venezuela, Carabobo, Cerro San Isidro, 1500–1600 m, 10.VIII.2003, T. Pyrcz *leg.*, prep. genit. 04/14.04.2016 K. Florczyk, CEP-MZUJ; ***Pseudomaniola phaselis argyritis*** (Thieme, 1907): 1 ♂: Bolivia, Cochabamba, Villa Tunari – Locotal, 1480–1500 m, 16.II.2009, T. Pyrcz & Y. Gareca *leg.*, CEP-MZUJ, prep. genit. 97/14.10.2016 A. Zubek; ***Junea doraete*** (Hewitson, [1858]): 1 ♀: Ecuador, Cord. Lagunillas, Jimbura – Laguna Negra, 15.V.1998, A. Jasiński *leg.*, prep. genit. 490/22.11.2016 J. Lorenc-Brudecka, CEP-MZUJ; ***Lasiophila cirta atropulla*** Pyrcz 2004: 1 ♀: Peru, Amazonas, Abra Pardo Miguel, 2200–2400, II.2003, prep. genit. 01/30.03.2016 K. Florczyk, CEP-MZUJ; ***Mygona irmina*** (E. Doubleday [1849]: 1 ♀: Venezuela, Aragua, Colonia Tovar, Los Colonos, 2100 m, 25.II.2007, T. Pyrcz *leg.*, prep. genit. 492/22.11.2016 J. Lorenc-Brudecka, CEP-MZUJ; ***Apexacuta astoreth*** (Thieme, 1907): 1 ♀: Peru, Apurimac, Distrito Abancay, Ampay, 3200 m, III.2005, J. Bottger *leg.*, prep. genit. 491/22.11.2016 J. Lorenc-Brudecka, CEP-MZUJ; ***Cheimas opalinus*** (Staudinger, 1897): 1 ♀: Venezuela, Trujillo, Guaramacal, Qda. El Caote, 2700–2750 m, 16.II.2006, T. Pyrcz *leg.*, prep. genit. 488/22.11.2016 J. Lorenc-Brudecka, CEP-MZUJ; ***Oxeoschistus cothon*** Salvin, 1871: 1 ♀: Costa Rica, Prov. San Jose, Cerro de la Muerte, Division–Sta Eduviges, 1900–2050 m, 13.03. 2016, 9°29′41″N, 83°44′04″W, T. Pyrcz *leg.*, DNA extraction number AZ–153, prep. genit. 365/11.04.2013 J. Lorenc-Brudecka, CEP-MZUJ.

## Results

### Molecular data

**Fig 1:**
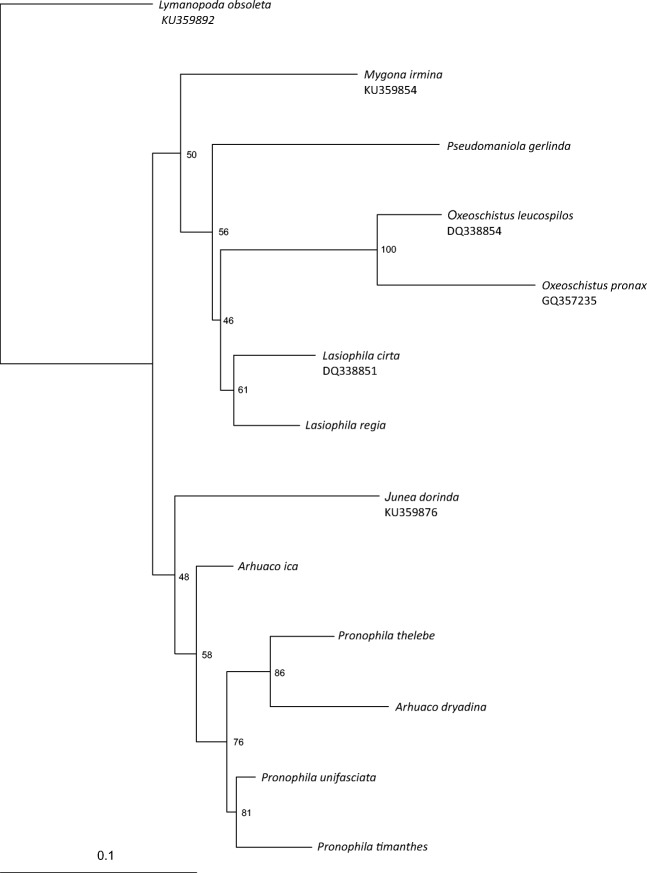
RAxML phylogenetic tree based on the COI gene. Bootstrap values are indicated at the nodes.

**Fig 2:**
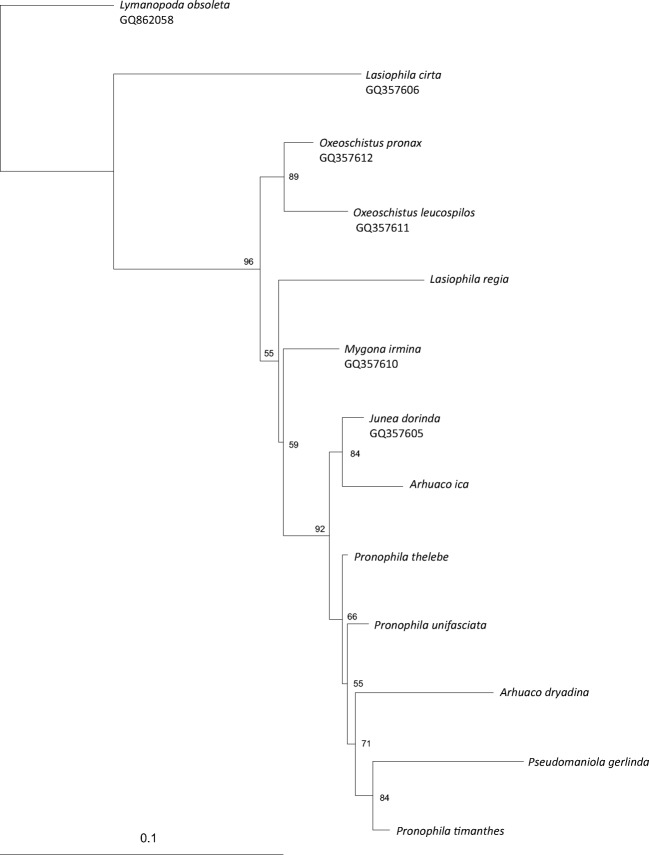
RAxML phylogenetic tree based on the *RpS5* gene. Bootstrap values are indicated at the nodes.

**Fig 3:**
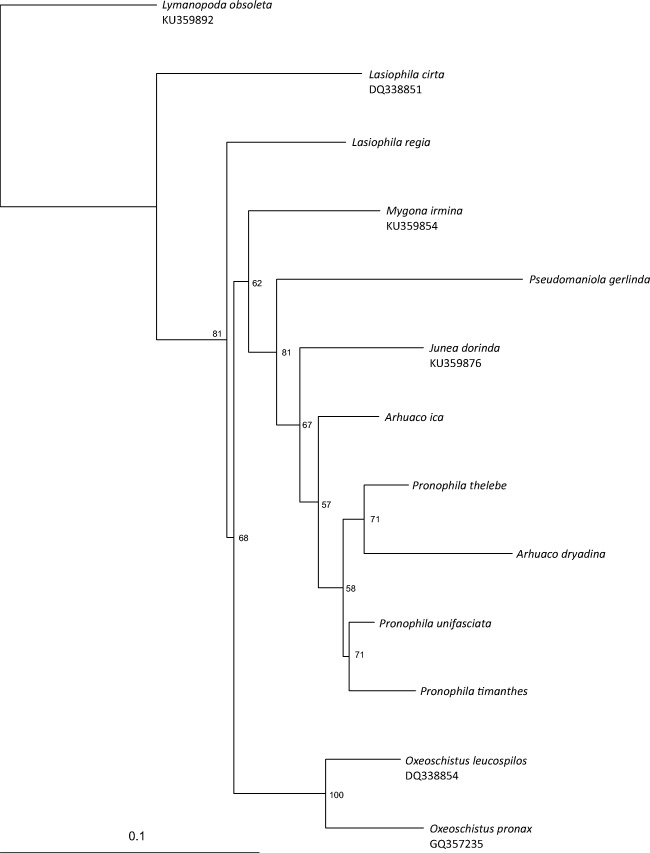
RAxML phylogenetic tree based on the joined *COI* and *RpS5* genes. Bootstrap values are indicated at the nodes.

The genetic pairwise distance between analyzed *COI* sequences ranged from 3.3 to 13.9%, and from 1.2 to 15.3% between *RpS5* gene sequences. The genetic distance between *Arhuaco* and *Pronophila* was below 7% (Tables 1, 2). Analysis of *COI* resulted in two sister clades, with one grouping three species of *Pronophila*, *A. ica*, *A. dryadina*, and *Junea dorinda* (Felder & Felder, 1862), and the second clade consisting of other four genera of Pronophilina (Fig 1). The phylogenetic tree based on *RpS5* indicated an internal position of *P. gerlinda* and *A. dryadina* within the *Pronophila* clade, and a sister species position of *A. ica* relative to *J. dorinda* (Fig 2). The concatenated tree showed the clade comprising *Pronophila* + *Arhuaco*, with *A. dryadina* as sister species to *P. thelebe* (Fig 3).

**Table 2:** Genetic pairwise distances between analyzed *RpS5* sequences.

*Mygona irmina*_GQ357610												
*Junea dorinda*_GQ357605	0.049											
*Lasiophila cirta*_GQ357606	0.136	0.128										
*Oxeoschistus leucospilos*_GQ357611	0.047	0.052	0.128									
*Oxeoschistus pronax*_GQ357612	0.041	0.052	0.122	0.031								
*Lymanopoda obsoleta*_GQ862058	0.105	0.115	0.124	0.124	0.111							
*Pronophila thelebe*	0.043	0.019	0.126	0.049	0.045	0.111						
*Pronophila unifasciata*	0.047	0.027	0.128	0.058	0.052	0.113	0.012					
*Pronophila timanthes*	0.047	0.027	0.128	0.054	0.045	0.115	0.012	0.014				
*Arhuaco dryadina*	0.082	0.058	0.151	0.085	0.085	0.134	0.047	0.052	0.045			
*Pseudomaniola gerlinda*	0.072	0.052	0.153	0.078	0.070	0.140	0.037	0.039	0.025	0.070		
*Lasiophila regia*	0.045	0.052	0.136	0.052	0.054	0.122	0.047	0.056	0.047	0.076	0.072	
*Arhuaco ica*	0.058	0.027	0.134	0.068	0.064	0.120	0.029	0.029	0.033	0.064	0.058	0.066

### Comparative morphology

**Fig 4:**
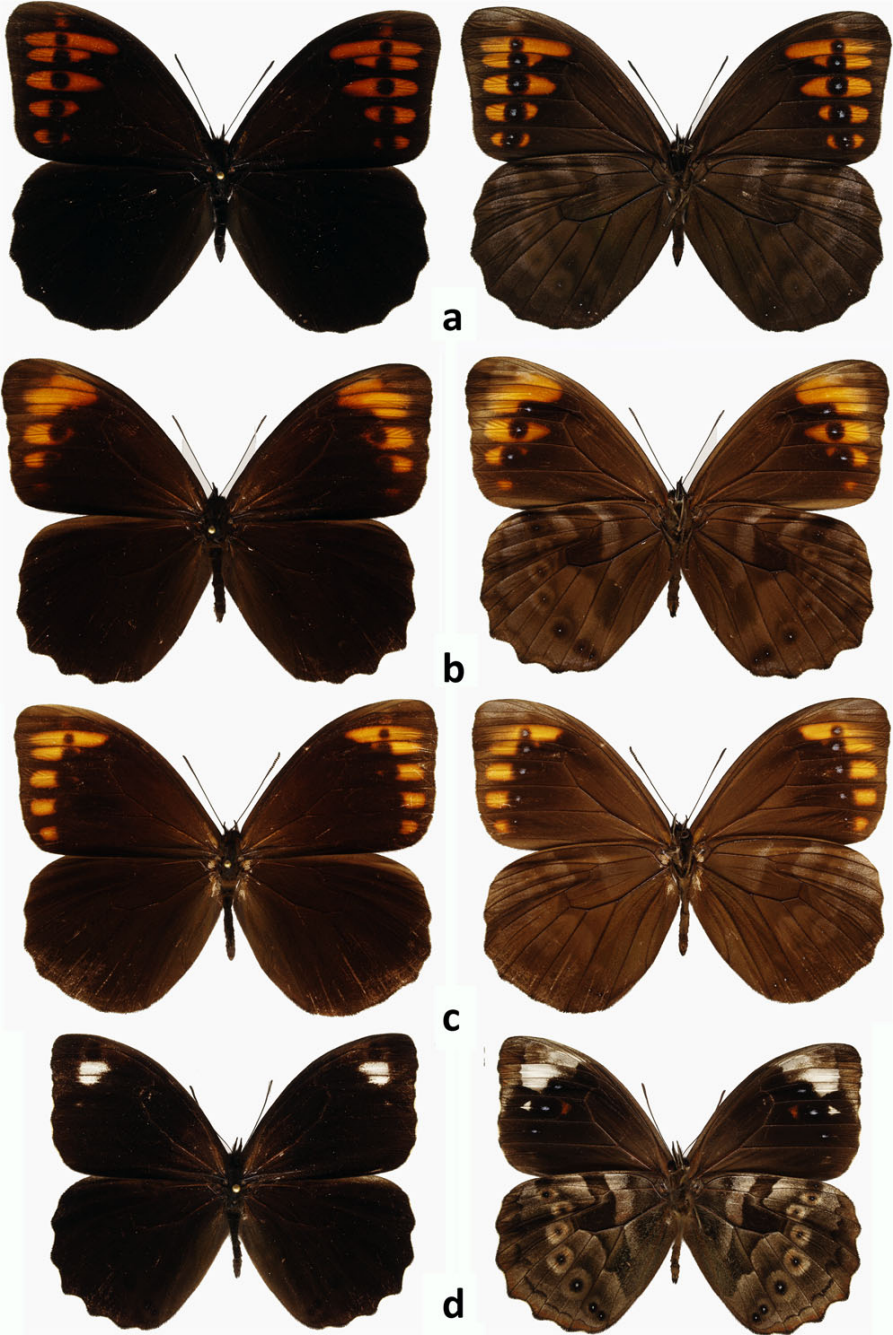
*Pronophila* adults, males (recto, verso): **a***Pronophila timanthes*, **b***Pronophila unifasciata unifasciata*, **c***Pronophila thelebina thelebina*, **d***Pronophila thelebe.*

The genus *Pronophila* is morphologically exceptionally homogenous (Fig 4). In addition, its head morphology (hairy eyes, slender antennae approximately two-fifths length of costa gradually widening into a weakly marked club, proportions of the segments of labial palps), venation pattern (long forewing discal veinlet, a well-developed hindwing humeral vein, and cross cu1–cu2 vein bent into discal cell), androconia (very large forewing dorsal androconial patch, covering two thirds of wing surface in median area), male genitalia (stout uncus, long and thin subunci, valva narrowing gradually towards apex with smooth dorsal surface without any dorsal processes and short, straight, tubular aedeagus), and female genitalia (oval bursa with prominent signa, large membraneous pocket enclosing antrum, a single slat-like sclerotized postvaginal process) do not present any consistent differences compared with other genera of the so-called large Pronophilina clade, including *Junea* Hemming 1964, *Eteona* Doubleday 1848, *Foetterleia* Viloria 2004, *Daedalma* Hewitson 1858, *Oxeoschistus* Butler 1867, *Proboscis* Thieme 1907, *Lasiophila* C. Felder & R. Felder 1859, *Thiemeia* Weymer 1911, *Apexacuta* Pyrcz 2004, *Corades* Doubleday [1849] and *Pseudomaniola* Röber 1889 (excluding some species of the latter two genera whose monophyly is not yet resolved) (Peña *et al*^2006^, Pyrcz 2010), and also *Arhuaco*.

At the same time, the species of *Pronophila* are immediately recognized from other genera of this group, including *Arhuaco*, by a series of characters of wing shape, namely, forewing with a blunt apex and gently concave outer margins; rounded hindwing wider than forewing with wavy outer margins; between three and five large, black forewing ventral submarginal ocelli (in species with white subapical patches the subapical ocelli are subdued) invariably with violet or blue pupils, aligned in a row either parallel to the outer margin or slightly arched basally; and hindwing venter median band darker than the rest of wing’s surface, continuous, with a nearly straight basal edge and a distal edge sharply produced distally along discal cell edge.

**Fig 5:**
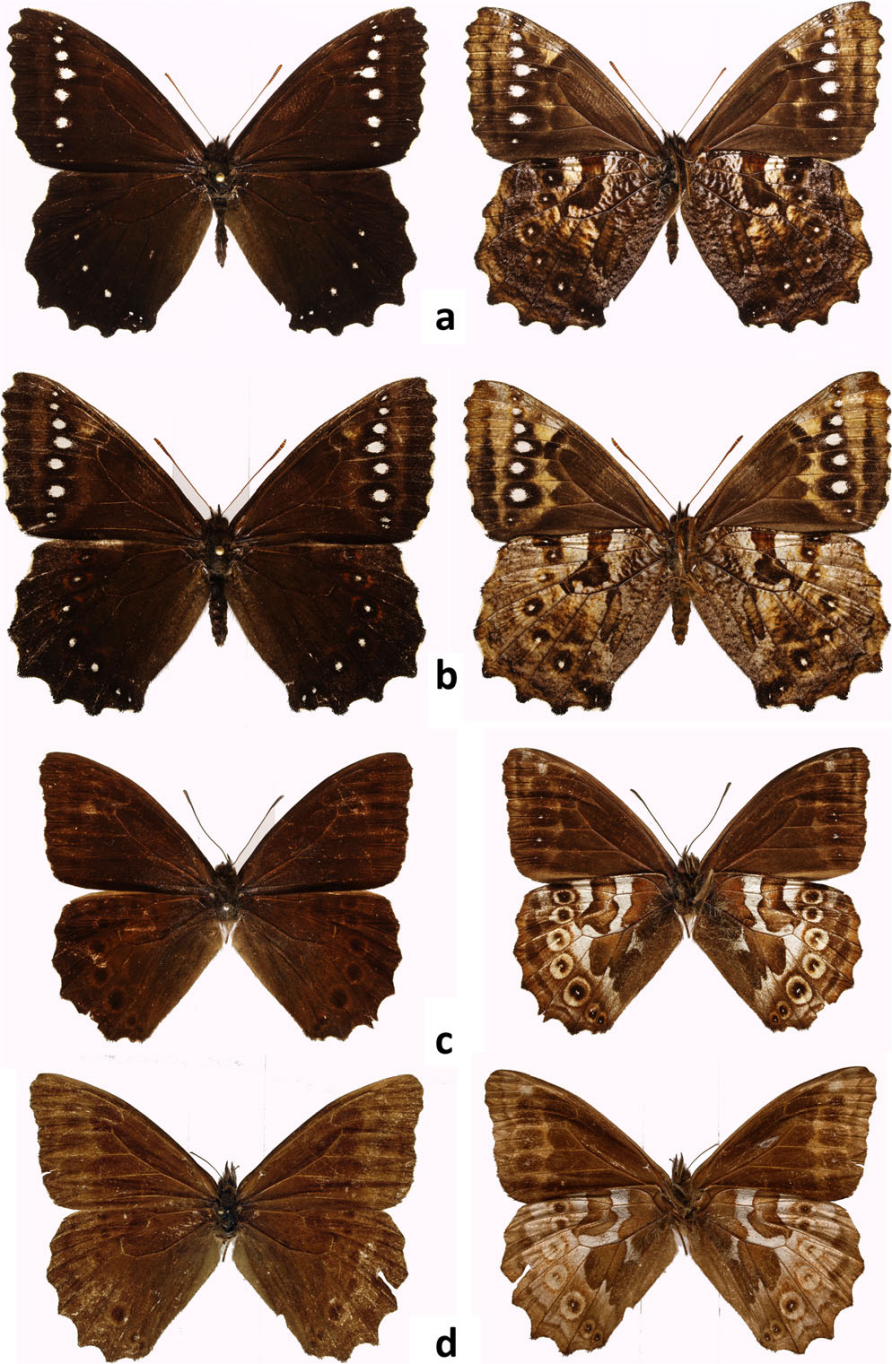
*Arhuaco* adults (recto, verso): **a***Arhuaco dryadina* male, **b***Arhuaco dryadina* female, **c***Arhuaco ica* male, **d***Arhuaco ica* female.

**Fig 6:**
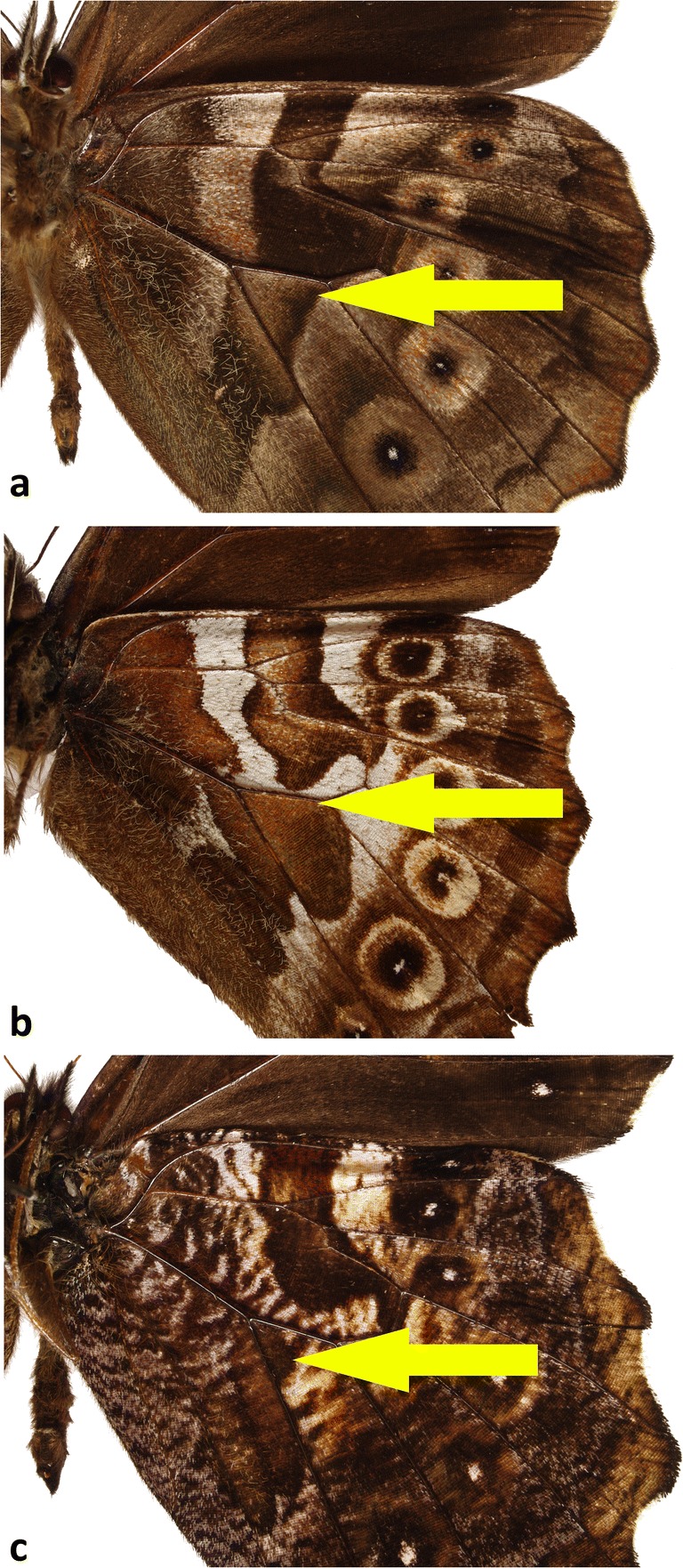
Hindwing ventral color patterns (arrows indicating the distortion of the Nymphalid ground-plan in *Arhuaco* consisting in the breaking and basal displacement of the postdiscal line—the so-called Pierellization): **a***Pronophila colocasia*, **b***Arhuaco ica*, **c***Arhuaco dryadina.*

In *Arhuaco* (Fig 5), in contrast to *Pronophila*, the forewing has an acute apex and straight outer margin, the hindwing is subtriangular with a scalloped outer margin with sharp tips at vein ends (in this respect, the wing shape of *Arhuaco* strongly resembles that of *Pseudomaniola*, as a result of which *A. dryadina* has been previously associated with this genus). Importantly, on the hindwing venter, the median band is discontinuous in the discal cell along cross vein Cu_A_1–Cu_A_2, with its basal edge connected to the postbasal line (Fig 6). Additionally, on the forewing underside, there is a row of black submarginal ocelli roughly parallel to the outer margin (in *A. ica*, the ocelli do have small violet pupils as in *Pronophila*, whereas in *A. dryadina* the pupils are white and very large; in *A. ica*, there are five well-developed ocelli and two in the apex transformed into whitish patches; in *A. dryadina*, there are as many as eight developed ocelli, including in the apical area in cells R3–R4 and R4–R5, which is more than in any other genus of Pronophilina). Finally, there is a row of hindwing black ocelli ringed with red on wing dorsal surface, most prominently in the females.

**Fig 7:**
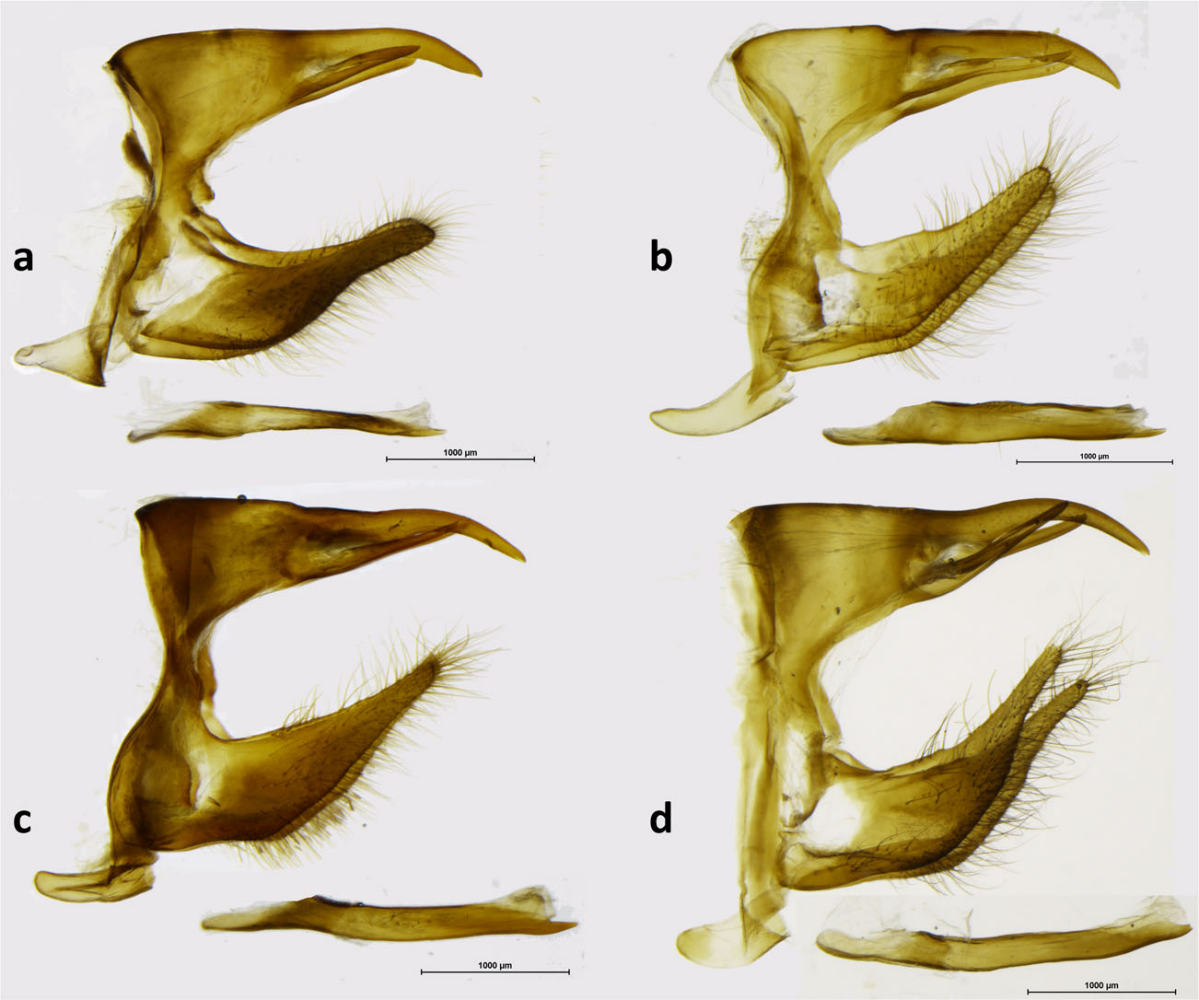
Male genitalia (lateral view, aedeagus extracted): **a***Arhuaco dryadina*, **b***Arhuaco ica*, **c***Pronophila orcus*, **d***Pronophila timanthes.*

The male genitalia of *Arhuaco* are very simple and do not present any distinctive character compared to *Pronophila* or *Junea*. All these three genera present a stout uncus (always shorter than in *Oxeoschistus*) aligned with the tegumen shoulder, long and thin subunci, the valva narrowing gradually towards the apex with a smooth dorsal surface (contrary to the serrate dorsal surface in *Oxeoschistus*) without any dorsal processes, and a short, and straight, tubular and smooth aedeagus (Fig 7).

### Female of *Arhuaco dryadina*

**Fig 8:**
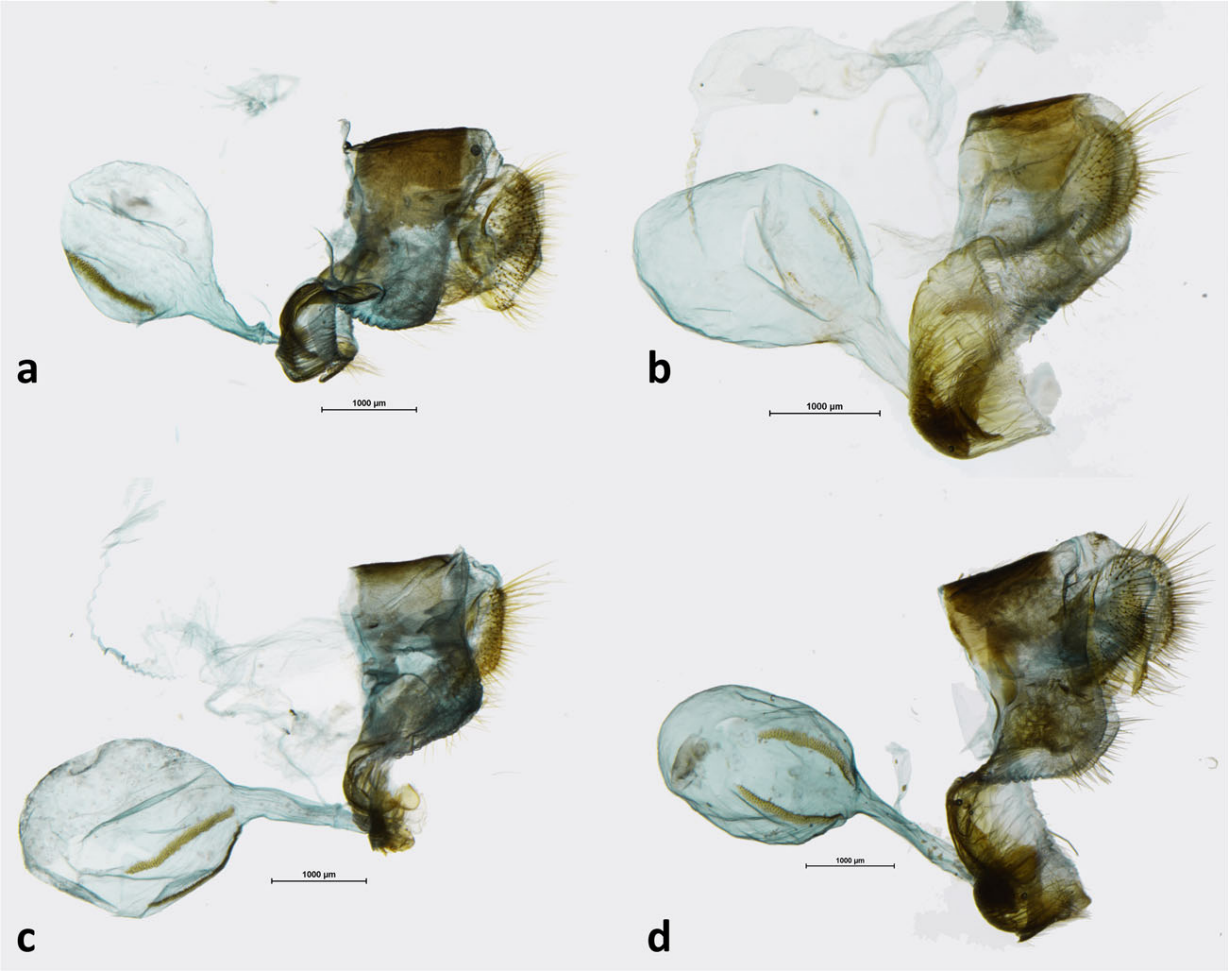
Female genitalia (lateral view): **a***Arhuaco dryadina*, **b***Arhuaco ica*, **c***Pronophila rosenbergi*, **d***Pronophila timanthes.*

Sexual dimorphism of *A. dryadina* is slight (Fig 5). The female is marginally larger (~ 2 mm in wingspan) than the male. On the upperside, its brown ground color is a shade lighter, and the white submarginal ocellar elements on the fore and hindwing are larger, bordered with a light brown halo, and on the hindwing additionally ringed with red. The underside ground color is lighter, predominantly sandy yellow, except for the basal half on the forewing which is brown with a light yellowish suffusion. The female genitalia (Fig 8) are characterized by an oval bursa with prominent signa, a large membranous pocket enclosing the antrum, and a single slat-like sclerotized postvaginal process.

### Diagnosis of *Arhuaco*

The original generic diagnosis of *Arhuaco* (Adams & Bernard 1977) does not stand in the light of the above comparative study. Consequently, a new diagnosis of the genus *Arhuaco* is presented as follows:

Adults: Forewing with an acute apex (blunt in *Pronophila* and *Mygona*) and straight outer margin (produced below apex in *Pseudomaniola* and *Junea*); hindwing subtriangular with a scalloped outer margin with sharp tips at vein ends (similar to *Junea*); hindwing venter postdiscal band discontinuous in discal cell along cross vein Cu_A_1–Cu_A_2, with basal edge connected to postbasal line (unlike any other genus of Pronophilina); forewing underside with a row of five to eight black submarginal ocelli roughly parallel to the outer margin (similar to *Pronophila*), including two, apical in R3–R4 and R4–R5 (unlike any other genus of Pronophilina), transformed into whitish patches in *A. dryadina*; black hindwing ocelli ringed with red visible on wing dorsal surface (unlike any other genus of Pronophilina). Sexual dimorphism slight, females larger, with lighter hindwing ventral patterns and hindwing ocelli more prominently marked on dorsal surface.

### Bionomics

During this research, *A. dryadina* was found in a rather flattened part of the uppermost area of a mountain ridge, with high and predominantly uniform forest cover dominated by oaks (*Quercus costarricensis*) at 3000–3100 m a.s.l. Individuals are very actively patrolling. Both males and females fly rather rapidly, but not as fast as some other Andean satyrines, such as *Corades*. On the wing, they resemble somewhat some species of *Pronophila* or *Pseudomaniola*, with the difference that they move invariably high above the ground, usually in the canopy, compared to other Andean or Mesoamerican satyrines which can be observed in the subcanopy but frequently descend to ground level. Perching coupled with territorial behavior among the Pronophilina satyrines is typical of very fast flying species in *Corades* and *Junea*, but is also present in some *Steremnia* Thieme, *Daedalma*, and *Thiemeia*. Other genera are either patrolling, or they present intermittent patrolling and perching behavior with no strong territoriality. Individuals, mostly males, observed in Cerro de la Muerte were very active, starting from 8 A.M. when the day was bright from the early morning. When days were mostly cloudy, they started being active during short sunny intervals almost immediately. For most of the time, they were the only active butterflies in the forest, as other species were apparently awaiting an increase in temperature, requiring at least 10 min of direct sunshine before taking flight. Males displayed patrolling behavior. They flew vigorously, high above the trees, occasionally fluttering and approaching some leaves or flowers, although never settling. They showed an obvious preference for *Quercus* trees, and, rather surprisingly, they rarely approached the abundant and frequently tall bamboo clumps, contrary to what can be observed with most of Andean satyrines, which are most frequently associated with their host plants. They were not attracted to traps placed at 1–2 m baited with dog excrement, which otherwise proved effective with other local satyrines. They were never observed on hilltops or other prominent topographical features, such as outstanding trees, contrary to territorial *Corades* and *Junea*, or non-territorial *Steremnia, Pedaliodes* or several other genera in the Andes. Male individuals were seen engaging in short interactions consisting of following one another for a short while, probably identifying whether the followed individual was a female, but no contest behavior was observed. No courtship or mating was observed. Individuals of *A. dryadina* spent most of the time in the canopy and were extremely reluctant to approach ground level, which was observed only above a boggy clearing covered partially with *Vaccinium* and *Hypericum* bushes. Even then, individuals overflew the plants but never approached within 2 m of the ground. DeVries (1987) reports *A. dryadina* as a rare butterfly. This is certainly not the case in the Cerro de la Muerte, where by the end of June and beginning of July *A. dryadina* was arguably common, and also the most obvious butterfly considering it was very active, patrolling, and a large butterfly that could unlikely be confused with any other species. In February–March (dry season), only one individual of *A. dryadina* was observed in the same area where they were common in June–July (rainy season), which indicates that the species is possibly strongly seasonal. Such important seasonal fluctuation in abundance is uncommon among tropical Pronophilina and has been reported so far only for species occurring in páramo grassland, for example, *Steromapedaliodes* (Pyrcz *et al*^2017^). The early stages and host plants are not known.

The only information on the ecological preferences of *A. ica* was published by Adams & Bernard (1977). They stated that the species is local and mostly rare, and that it has a preference for forest canopy, and rarely descends to the ground. One of the co-authors, however, observed and collected males mud-puddling on the shores of a mountain stream, which shows that occasionally they descend to ground level. *Arhuaco ica* has been collected so far at 2400–3100 m a.s.l., therefore in upper elevation cloud forests. The early stages of *A. ica* are not known, but its host plants are almost certainly among *Chusquea* bamboos, as for other species of high elevation Pronophilina in the Sierra Nevada de Santa Marta.

## Discussion

The currently available morphological and genetic data resulted in incongruent inferences about the relationships among the studied species. In addition, the two sequenced genes COI and RpS5 also yielded different results, segregating either the two species of *Arhuaco* within the *Pronophila* clade, placing *Arhuaco ica* as sister to *Pronophila* + *Arhuaco dryadina* or indicating *A. ica* as sister to *J. dorinda*, and even placing *Pseudomaniola gerlinda* within the *Pronophila* clade. Although only two genetic markers were studied here, the phylogenies within this section of Pronophilina seem unresolved, and the monophyly of *Arhuaco* should be considered as uncertain. Interestingly, however, the molecular results strongly indicate a clade composed of *Pronophila*, *Arhuaco*, *Junea*, and *Pseudomaniola*, and, if adopting a broader taxonomic approach, all these could be lumped within a larger genus *Pronophila*. These results, incidentally, agree with morphological evidence which indicates close relationships among these genera, possibly forming a monophyletic group.

Putative synapomorphies, especially in the wing shape and color pattern involving the so-called elements of the nymphalid groundplan sensu Schwanwitsch (Nijhout 1991), suggest a closer relationship of *A. ica* with *A. dryadina* than with any other genus or species of the subtribe Pronophilina and indicate that *Arhuaco* is indeed monophyletic. The broken median band with the basal edge connected to the postbasal line is a rare distortion of the nymphalid groundplan termed “Pierellization” by Schwantwitsch, described for the Haeterini genus *Pierella* (Nijhout op. cit.). Such a Pierellization in the two species of *Arhuaco* is a unique state in any genus or species of Pronophilina and was considered to be a strong generic synapomorphy (Pyrcz 2004). On the other hand, the two species do not present any apparent putative synapomorphic characters enabling to associate them with the genus *Pronophila*, presenting rather some features of other genera, in particular of *Junea* and *Pseudomaniola.*

Ecological and behavioral data also indicate closer affinities between *A. ica* and *A. dryadina* than with any species of *Pronophila*. They both occur in the same kind of habitat, high elevation forests, at higher elevations than *Pronophila*, usually close to the upper forest limit. They also both have an unusual predilection among the Pronophilina for flying in the high canopy and not necessarily in association with bamboo thickets, in contrast to *Pronophila* which are invariably found in the vicinity of bamboo and generally in the forest understory.

At this stage, there is not enough evidence to ascertain the monophyly of *Arhuaco*, since it is not supported by molecular data. It would be, however, premature to synonymize *Arhuaco* with *Pronophila* given sharp-cut morphological differences between the two. More data are needed, in particular the use of more genetic markers, and the inclusion of even more species of *Pronophila* as well as other species of *Junea* and *Pseudomaniola* in the analysis. It is possible that early-stage data would shed some light on the affinities of *Arhuaco* and *Pronophila*, as they have on the status of the genus *Daedalma*, which has very particular larvae compared to other related genera (Pyrcz *et al*^2011^), but given the scarcity of *Arhuaco ica* in the field, and the difficulties in finding pronophiline larvae, and rearing them, requiring in situ work, it will be a very difficult task.

The disjunct distribution of *Arhuaco* in Costa Rica–Panama and northern Colombia, if the genus is confirmed as monophyletic, would be challenging to explain from a biogeographical and evolutionary perspective. Recent, long-distance dispersal of such highly sedentary and habitat-specific montane satyrines between these areas that are currently separated by the vast Colombian plains seems extremely unlikely (Adams 1986, Pyrcz & Garlacz 2012). Therefore, a vicariance scenario has to be considered. In this case, *Arhuaco* would originally have had a wider distribution, but disappeared from intervening areas in the northern Andes of Colombia. Such a vicariance scenario would imply that *Arhuaco* is an old genus within the subtribe which perhaps emerged prior to the radiation of the genus *Pronophila*. This hypothesis, in turn, could explain, to some extent, the closer genetic relationships with the latter genus and the position of *Arhuaco* within the larger *Pronophila* clade.

However, we have to consider that the origins of *Arhuaco* and, in fact, of other montane species of butterflies belonging to predominantly Andean clades in Central America are presumably linked with the formation of the Isthmus of Panama, and the process of the Great Interamerican Biotic Interchange (GABI). It is generally considered that the land bridge connection between South and Central America occurred only some 3 MYA, and before that higher mountainous areas in Central America were isolated volcanic islands, although some data indicate that such a connection could have existed some 10–15 MYA (Montes *et al*^2015^). The formation of a land connection between these two areas was a precondition of colonization, but, additionally, ecological conditions must have evolved to allow montane species from South America to establish themselves in Central American highlands. This, in turn, could have happened with the cooling of global climate during the Pleistocene and the evolution of ecological corridors of suitable vegetation, hence permitting dispersal from the Andes to the Cordillera de Talamanca. In any case, the timing of such a scenario does not predate the Pleistocene, which is some 1.85 MYA. It is congruent with the distribution of most Central American species of Pronophilina, an overwhelming majority of which are closely related to north Andean taxa, including *Lymanopoda euopis* Godman & Salvin, *Pedaliodes ereiba* Godman & Salvin, *Pedaliodes lithochalcis* Butler & Druce, *Eretris suzannae* DeVries, or *Pronophila timanthes* Salvin (Casner & Pyrcz 2010, Pyrcz & Viloria 2012). These are mostly mid- to low-altitude species whose ancestors presumably dispersed relatively recently from the Andes. However, the presence of the high elevation specialist *A. dryadina*, and indeed of the endemic Mesoamerican genus *Drucina* represented by one Costa Rican species *D. leonata* Butler 1872, and by *D. championi* (Godman & Salvin, [1881]) from Guatemala and southern Mexico, is difficult to explain by geological and paleoclimatic data on the one hand, and species phylogenetic affinities and the inferred timing of divergence on the other.
